# 5-HT_2_ and 5-HT_7_ receptor agonists facilitate plantar stepping in chronic spinal rats through actions on different populations of spinal neurons

**DOI:** 10.3389/fncir.2014.00095

**Published:** 2014-08-19

**Authors:** Urszula Sławińska, Krzysztof Miazga, Larry M. Jordan

**Affiliations:** ^1^Department of Neurophysiology, Nencki Institute of Experimental Biology PASWarsaw, Poland; ^2^Department of Physiology, Spinal Cord Research Centre, University of ManitobaWinnipeg, MB, Canada

**Keywords:** locomotion, recovery, spinal cord, total transection, serotonin

## Abstract

There is considerable evidence from research in neonatal and adult rat and mouse preparations to warrant the conclusion that activation of 5-HT_2_ and 5-HT_1A/7_ receptors leads to activation of the spinal cord circuitry for locomotion. These receptors are involved in control of locomotor movements, but it is not clear how they are implicated in the responses to 5-HT agonists observed after spinal cord injury. Here we used agonists that are efficient in promoting locomotor recovery in paraplegic rats, 8-hydroxy-2-(di-n-propylamino)-tetralin (8-OHDPAT) (acting on 5-HT_1A/7_ receptors) and quipazine (acting on 5-HT_2_ receptors), to examine this issue. Analysis of intra- and interlimb coordination confirmed that the locomotor performance was significantly improved by either drug, but the data revealed marked differences in their mode of action. Interlimb coordination was significantly better after 8-OHDPAT application, and the activity of the extensor soleus muscle was significantly longer during the stance phase of locomotor movements enhanced by quipazine. Our results show that activation of both receptors facilitates locomotion, but their effects are likely exerted on different populations of spinal neurons. Activation of 5-HT_2_ receptors facilitates the output stage of the locomotor system, in part by directly activating motoneurons, and also through activation of interneurons of the locomotor central pattern generator (CPG). Activation of 5-HT_7/1A_ receptors facilitates the activity of the locomotor CPG, without direct actions on the output components of the locomotor system, including motoneurons. Although our findings show that the combined use of these two drugs results in production of well-coordinated weight supported locomotion with a reduced need for exteroceptive stimulation, they also indicate that there might be some limitations to the utility of combined treatment. Sensory feedback and some intraspinal circuitry recruited by the drugs can conflict with the locomotor activation.

## Introduction

During the last few decades the potential role of 5-HT in locomotor system activation, modulation and functional recovery after spinal cord lesions has received considerable attention and continues to be an area of major interest and investigation (reviewed in Hochman et al., 2001; Jordan and Schmidt, 2002; Orsal et al., 2002; Jordan and Sławińska, 2011). Using the *in vitro* neonatal rat spinal cord preparation it was demonstrated that 5-HT application induces locomotor-like discharge of lumbar ventral roots (Kudo and Yamada, 1987; Cazalets et al., 1990, 1992; Sqalli-Houssaini et al., 1993; Cowley and Schmidt, 1994; Kiehn and Kjaerulff, 1996), the frequency of which was concentration-dependent (Cazalets et al., 1992; Beato et al., 1997). The selective application of 5-HT on different spinal cord levels in this preparation using the bath partitioning method confirmed that the 5-HT-sensitive oscillatory network, capable of producing a locomotor-like pattern of activity, is diffusely distributed throughout the supralumbar spinal cord and mediates descending rhythmic drive to lumbar motor centers (Cowley and Schmidt, 1994, 1997; Schmidt and Jordan, 2000). The lumbosacral region in isolation is responsible for inducing only the tonic discharge that, at least in part, could be related to the direct excitatory effect of 5-HT on lumbar motoneurons. There are further data suggesting a regional differentiation of certain receptors in different rostro-caudal regions (Jordan and Schmidt, 2002; Liu and Jordan, 2005; Liu et al., 2009) that are in line with the differential effects of 5-HT on supralumbar *vs*. lumbar regions. There is increasing evidence that activation of specific serotonin receptors in the spinal cord is effective for the production of locomotor activity (Antri et al., 2005; Sławińska et al., 2012b). A number of serotonergic receptors are found in the spinal cord, but 5-HT_2A_, 5-HT_2C_ and 5-HT_7_ are the major ones implicated in the control of locomotion (Schmidt and Jordan, 2000; Hochman et al., 2001; Liu and Jordan, 2005; Landry et al., 2006; Liu et al., 2009; Dunbar et al., 2010; Fouad et al., 2010; Bos et al., 2013). Attempts to restore locomotion after spinal cord injury using systemic drug applications, combined with other interventions such as training and electrical stimulation of the spinal cord, have included the use of agents that target these receptors (Courtine et al., 2009; Musienko et al., 2011; van den Brand et al., 2012). The most commonly used agonists that are effective when applied systemically are quipazine, which has high affinity for both 5-HT_2A_ and 5-HT_2C_ receptors, and 8-hydroxy-2-(di-n-propylamino)-tetralin (8-OHDPAT), which binds selectively to 5-HT_7_ and 5-HT_1A_ receptors (Antri et al., 2003; Landry et al., 2006; Sławińska et al., 2012b).

Because of the strong evidence suggesting that serotonergic innervation that descends from brainstem raphe nuclei to the spinal cord is involved in activation and modulation of central pattern generator (CPG) activity (Schmidt and Jordan, 2000; Jordan and Schmidt, 2002; Liu and Jordan, 2005; Liu et al., 2009), increased attention to the use of intraspinal grafting of 5-HT neurons to enhance restoration of locomotion is warranted (Orsal et al., 2002; Sławińska et al., 2014). It was demonstrated that in paraplegic rats with the serotonergic innervation in the spinal cord destroyed by total transection, sublesional transplantation of embryonic brainstem tissue containing 5-HT neurons is able to enhance the recovery of locomotor hindlimb movements (Feraboli-Lohnherr et al., 1997; Ribotta et al., 2000; Sławińska et al., 2000, 2013; Majczyński et al., 2005). In our recent paper we demonstrated for the first time that restored coordinated hindlimb locomotion by this grafting technique is mediated by 5-HT_2_ and 5-HT_7_ receptors (Sławińska et al., 2013). Moreover we demonstrated that it acts through different populations of spinal locomotor neurons. Specifically, 5-HT_2_ receptors control CPG activation as well as motoneuron output, while 5-HT_7_ receptors contribute primarily to activity of the locomotor CPG. These results are consistent with the roles for these receptors during locomotion in intact rodents and in rodent brainstem-spinal cord *in vitro* preparations (Madriaga et al., 2004; Liu and Jordan, 2005; Pearlstein et al., 2005; Liu et al., 2009; Dunbar et al., 2010).

Because the activation of these receptors is currently a prominent feature in the translational strategies that are now being undertaken to restore locomotion after spinal cord injury (Antri et al., 2005; Courtine et al., 2009; van den Brand et al., 2012), it is important to determine the mechanisms of action of these drugs on the spinal locomotor CPG and the output elements of the locomotor system, including motoneurons. At this point, there is little known about which neurons in the spinal locomotor networks and what functional components of the locomotor system are influenced by these drugs. Here we investigated these features of the actions of these drugs in rats with a chronic complete spinal cord injury. Motor performance on a treadmill was tested before and after i.p. drug application. Exteroceptive stimulation was used to trigger hindlimb movements that were monitored using video recordings synchronized with simultaneous electromyographic (EMG) recordings from the soleus and tibialis anterior muscles of both hindlimbs. In our previous paper using grafts of 5-HT neurons and serotonergic antagonists (Sławińska et al., 2013), we suggested the hypothesis that transplant-mediated activation of 5-HT_2_ receptors likely governs the function of both CPG neurons and motoneurons, whereas the 5-HT_7_ receptor activation may be more restricted to CPG neurons. Here we compare the effects of agonists to these receptors to test this hypothesis, using EMG recordings to determine the effects of these drugs on motoneuron activity and on inter- and intralimb coordination in chronic spinal rats.

A preliminary report of these results has been presented in an abstract form (Sławińska et al., 2011).

## Materials and methods

Experiments were performed on WAG (Wistar Albino Glaxo) female rats (*n* = 11) 3-months-old at the time of spinal cord injury. All surgical and experimental procedures were conducted with care to minimize pain and suffering of animals in accordance with the guidelines of the First Local Ethics Committee in Poland, according to the principles of experimental conditions and laboratory animal care of European Union, the Polish Law on Animal Protection, and of the University of Manitoba Animal Care Committee, in accordance with the guidelines of the Canadian Council on Animal Care.

### Spinal cord transection procedure

Complete spinal cord transection (SCI) was performed under deep anesthesia (Isofluorane, 2% and Butomidor, 0.05 mg/kg b.w.). Under aseptic conditions a mid-dorsal skin incision was performed at the rat back over Th8-11 vertebrae, and the back muscles were separated from the vertebral column. Then after laminectomy the spinal cord was completely transected between Th9/Th10 spinal cord level. A 2–3 mm piece of spinal cord tissue was cut using scissors and gently aspirated as previously described (Sławińska et al., 2000, 2012b; Majczyński et al., 2005). The muscles and fascia overlying the paravertebral muscles were closed in layers using sterile sutures, and the skin was closed with stainless-steel surgical clips. After surgery, the animals received a non-steroidal anti-inflammatory and analgesic treatment (Tolfedine 4 mg/kg s.c.), and during the following days the animals were given antibiotics (5 days Baytril 5 mg/kg s.c. and 8 days Gentamicin 2 mg/kg s.c.). The bladder was emptied manually twice a day until the voiding reflex was re-established (about 7 days). Female rats were chosen for these experiments because of the relative ease with which the manual bladder emptying can be achieved.

### Implantation of EMG recording electrodes

Two months after spinal cord transection the animals were anesthetized with Equithesin (3.5 ml/kg i.p., for the mixture details see Sławińska et al., 2000) and bipolar EMG recording electrodes were implanted in the extensor soleus (Sol) and flexor tibialis anterior (TA) muscles of both hindlimbs as previously described (Sławińska et al., 2000, 2012b; Majczyński et al., 2005). The electrodes were made of Teflon-coated stainless-steel wire (0.24 mm in diameter: AS633, Cooner Wire, Chastworth, CA, USA). The hook electrodes (1.5 mm of the insulation removed) were inserted into investigated muscles and secured by a suture. The distance between the tips of electrodes was 1–2 mm. The ground electrode was placed under the skin on the back of the animal. After electrode implantation animals received a single dose of antibiotic (Baytril 5 mg/kg s.c.).

### Video and electromyographic recordings

Video and EMG recording started 3–5 days after electrode implantation. One minute recordings at speeds of 5 cm/s or 10 cm/s were taken before and at a few time points after drug application as described previously (Sławińska et al., 2000, 2012b; Majczyński et al., 2005). EMG recordings during locomotor-like hindlimbs activity of the SCI rats before and after drug application were filtered (0.1–1 kHz band pass), digitized and stored on computer (3 kHz sampling frequency) using the Winnipeg Spinal Cord Research Centre capture system. The EMG activity was simultaneously recorded and synchronized with video recordings, thus enabling the further off-line analysis of portions of EMG activity related to the best locomotor performance in every animal.

### Evaluation of hindlimb locomotion

The quality of plantar stepping was established based upon EMG analysis, as described previously (Sławińska et al., 2012b, 2013, 2014). The rats were suspended above a treadmill with the forelimbs and thorax placed on a platform above the moving belt, and tail pinch was used to elicit hindlimb movements. Stimulation of tail or perineal area afferents has been used for eliciting locomotion in cases of complete spinal cord transection (Meisel and Rakerd, 1982; Pearson and Rossignol, 1991; Rossignol et al., 2006; Etlin et al., 2010; Lev-Tov et al., 2010; Sławińska et al., 2012b), and has been used in all prior attempts to reveal locomotor recovery after 5-HT transplants (Feraboli-Lohnherr et al., 1997; Ribotta et al., 2000; Sławińska et al., 2000, 2013; Majczyński et al., 2005). The tail stimulus was adjusted by the experimenter to maximize the quality of plantar stepping. The salient features of plantar stepping as defined here were: (1) sustained soleus activity throughout the stance phase; (2) soleus burst duration related to step cycle duration; (3) brief TA activity of consistent duration; and (4) consistent intra- and interlimb coordination (Sławińska et al., 2014).

### Drug test

To examine the effects of 5-HT agonists on the hindlimb locomotor-like movements we started from evaluation of the pre-drug baseline performance. Then the evaluation of hindlimbs movements was carried out 15–30 min after drug application. Quipazine (0.25 mg/kg dissolved in saline with 10% of propylene glycol) and (±)-8-hydroxy-dipropylaminotetralin hydrobromide (8-OHDPAT) (0.2–0.4 mg/kg dissolved in saline) were used to activate 5-HT_2_ and 5-HT_1_/5-HT_7_ receptors respectively. During the combined treatment, both drugs were applied with the dose 0.1 mg/kg within a 20 min delay in between (the quipazine i.p. injection was followed by the 8-OHDPAT i.p. applications). Quipazine was always first, because the effect of 8-OHDPAT is usually shorter than that of quipazine. To avoid any training or drug accumulation effects the locomotor testing was carried out not more often than twice a week with at least 3–4 days break in between. Doses were determined on the basis of previous experiments using these drugs (Antri et al., 2003; Majczyński et al., 2005; Musienko et al., 2011; Sławińska et al., 2012b).

### Statistical analysis

The EMG pattern related to the best locomotor movement in each experimental condition was analyzed using custom software.^1^ The coordination between left and right TA (interlimb coordination) and between right Sol and right TA (intralimb coordination) was analyzed using polar plots as previously described (Zar, 1974; Batschelet, 1981; Kjaerulff and Kiehn, 1996; Cowley et al., 2005; Sławińska et al., 2013). In the polar plots the position of the vector at 0 or 360° reflects synchrony of analyzed EMG burst onsets, whereas 180° is equivalent to alternation. The length of the vector (***r***, ranging from 0 to 1) indicates the concentration of phase values around the mean, and the strength of coordination between muscle burst onsets. Rayleigh’s circular statistical test was used to determine whether the inter- and intra-limb coordination ***r***-values were concentrated, suggesting coupling of burst activity, or dispersed, indicating loss of hindlimb coordination. The relationships between the EMG burst duration and step cycle duration were determined using the regression line method. Values are reported here as mean ± SD (Standard Deviation). The normal distribution of the data was confirmed using a Shapiro-Wilk test. For comparison of two groups of results (after the high dose of quipazine and 8-OHDPAT alone) Student’s *t*-Test was used. For comparison of the pre-drug data with the results obtained in two experimental conditions (after the low dose of quipazine, then after a subsequent low dose of 8-OHDPAT) one-way repeated measures ANOVA followed by Tukey’s *post-hoc* test for multiple comparison was used (Prism, GraphPad Software, La Jolla, CA).

## Results

**Figure 1:**
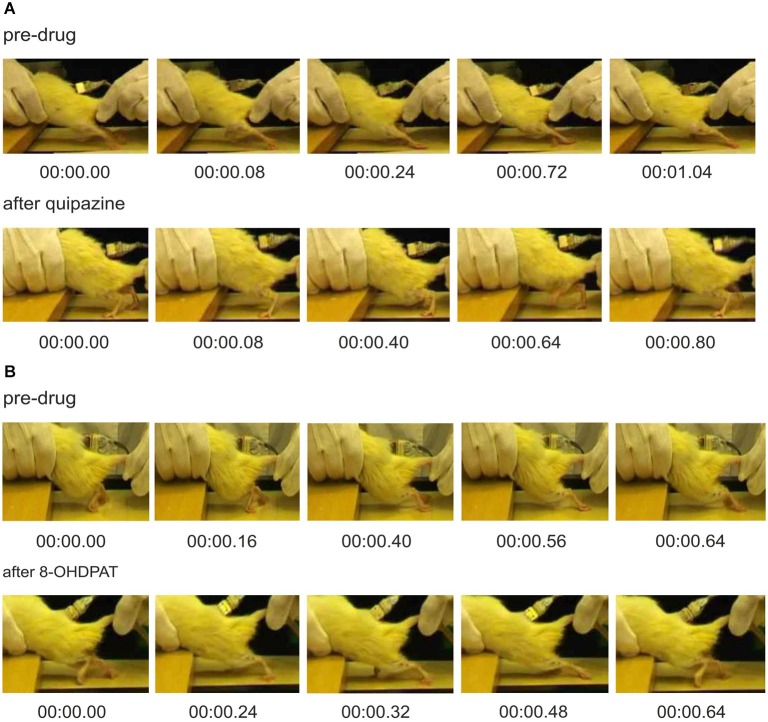
**Video frames showing consecutive phases of locomotor-like hindlimb movements in spinal rats before and after drug application (A) quipazine (0.25 mg/kg, i.p.) or (B) 8-OHDPAT (0.4 mg/kg, i.p.)**. Plantar paw placement and weight supported stepping are evident after application of either drug. However, after quipazine application, spinal rats present better body weight support (**A**, lower panel) in comparison to that after 8-OHDPAT (**B**, lower panel) where the hindlimbs remain extended behind the body.

Spinal cord total transection induced impairment of hindlimb movements in adult rats as described below. When inspected in their home cages the rats were typically paraplegic, and they used the forelimbs to move around with the hindlimbs dragging on the floor behind the body. Before drug application locomotor hindlimb movement of spinal rats was strongly impaired (Figures 1A,B upper panel). During locomotor trials on the treadmill the hindlimbs were kept extended behind the rat body and their movements were limited only to dragging of the paws with dorsal surfaces touching the moving treadmill belt. Tail stimulation induced some muscle contractions related to rather limited hindlimb movements, which were accompanied by uncoordinated EMG activity in antagonist Sol (antigravity extensor) and TA (dorsiflexor of the ankle joint) muscles, lack of rhythm consistency, and loss of sustained Sol muscle activity through the stance phase of the cycle. Moreover, the bursts of Sol muscle activity very often overlapped with ipsilateral TA muscle activity, often resulting in failure to initiate a normal swing phase of the step cycle. The details of locomotion induced by tail stimulation in spinal rats (Figure 1) have been described in our previous publications (Jordan and Sławińska, 2011; Sławińska et al., 2012b).

**Figure 2:**
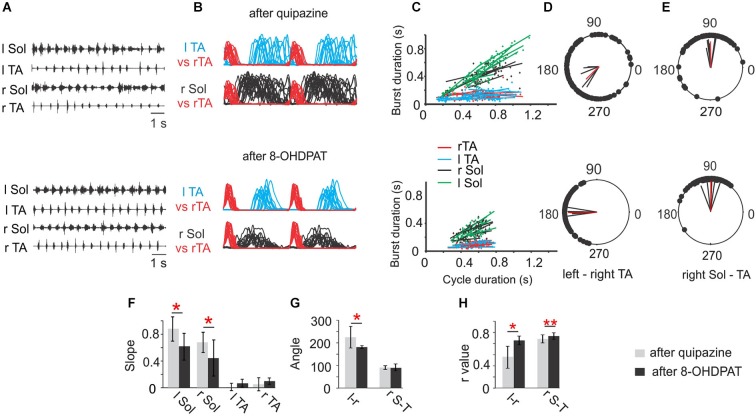
**Comparison of Soleus (Sol) and tibialis anterior (TA) EMG activity patterns during locomotor hindlimb movements enhanced by quipazine (upper panel) or 8-OHDPAT (lower panel) application**. **(A)** EMG activity during hindlimb movements in two experimental conditions (upper traces after quipazine; lower traces after 8-OHDPAT). **(B)** Envelops of EMG burst activity established from raw records rectified, filtered, and normalized to the step cycle (onset of activity in the right TA considered the onset of the cycle). Note longer burst activity of the soleus muscle during locomotor movements enhanced by quipazine application (upper panel) than after 8-OHDPAT (lower panel). **(C)** Regression lines illustrating the relationships between the step cycle durations and burst durations for the left and right TA and Sol muscles; **(D)** and **(E)** polar plots showing the relationships between the onset of right TA (r TA) activity and either the contralateral TA or the ipsilateral extensor (r Sol). The 0 position on the polar plot corresponds to the onset of activity in the right TA muscle, and the positions of the filled black circles indicate the times of onset of activity in the left TA—interlimb coordination (**D**, lower and upper panels) or the onset of activity in the right Sol—intralimb coordination (**E**, lower and upper panels), the black lines each represent results of analysis established for one animal and the red line represents the average results across animals in the analyzed group. **(F)** Bar diagrams showing the mean slopes (±SD) for the regression lines between step cycle durations and burst durations for the left and right TA and Sol muscles in rats in locomotor hindlimb movement enhanced by either drug. **(G)** Bar diagrams showing the means (±SD) of the angle of the left–right TA (l–r) and of the right Sol–right TA (r S–T) relationships (relative timing of the onsets for the two muscles) represented in each polar plot in locomotor movements enhanced by application of quipazine and 8-OHDPAT in spinal rats. **(H)** Bar diagrams showing the means (±SD) of ***r***-value between the times of onset of activity in both left-right and flexor-extensor EMG established for spinal rats treated either by quipazine or by 8-OHDPAT. Statistical significance by Student’s *t*-Test is indicated by red stars for (*n* = 6) spinal rats tested during locomotor movement enhanced by either drug: * *P* < 0.05; ** *P* < 0.005. The details of the comparison between pre-drug and each agonist alone were presented in Figures 2, 6 and 7 of Sławińska et al. (2012b).

We have also previously described improvement in locomotion produced by quipazine and 8-OHDPAT (Jordan and Sławińska, 2011; Sławińska et al., 2012b), but here we provide a comparison of the effects of these two agonists, and show how these data reveal aspects of the organization of the locomotor system that are influenced by serotonin receptor activation that have not previously been appreciated. A single dose of serotonin receptor agonists, quipazine or 8-OHDPAT, resulted in improvement of locomotor hindlimb movements in both cases (Figures 1A or 1B respectively). After i.p. injection of 0.25 mg/kg of quipazine, or 0.4 mg/kg of 8-OHDPAT, spinal rats could be induced by tail pinching to present regular hindlimb movements associated with plantar stepping. However, hindlimb locomotor movements observed after application of these two drugs were markedly different. Hindlimb movements were more regular after 8-OHDPAT than after quipazine application (confirmed by smaller Standard Deviation of the mean cycle duration; 536 ± 73 ms *vs*. 630 ± 210 ms). Moreover, after quipazine application, spinal animals presented better body weight support and extensor muscle activity in comparison to that after 8-OHDPAT. Improved locomotor hindlimb movements observed after application of both drugs were accompanied by sustained regular EMG recordings with alternating burst activity of extensor and flexor muscles. EMG activity was more regular than before drug application. In both cases EMG bursts of Sol muscle were robust during the period between ipsilateral flexor EMG bursts, which did not overlap with TA muscle bursts. EMG bursts of Sol and TA muscles were more regular and consistent after 8-OHDPAT than after quipazine application, but the excitation of Sol was more likely to be sustained throughout the stance phase after quipazine alone (Figures 2A,B, upper and lower panels). This is consistent with the absence of effective stance just before the onset of flexion observed in Figure 1B. Moreover, the burst duration of the Sol muscle was shorter during locomotion induced by 8-OHDPAT than after quipazine (duty cycle for soleus burst duration: 56 ± 10% *vs*. 72 ± 10%; Student’s *t*-Test, *P* < 0.05).

Investigation of the relationship between the EMG burst duration and step cycle duration using analysis of regression lines for left and right Sol and TA muscles show that after application of either 8-OHDPAT or quipazine, regression lines for Sol muscle burst duration plotted against cycle duration demonstrated a positive relationship (Figure 2C, upper and lower panels) that is not seen in control pre-drug analysis (for comparison see Sławińska et al., 2012b). It is important to note that after quipazine application the slopes of regression lines were significantly higher than after 8-OHDPAT administration (statistically significant, *P* < 0.05) suggesting that quipazine application leads to increased excitability of motoneurons or last-order interneurons (Figure 2F).

Using polar-plot analysis we investigated the inter- (left-right TA; Figure 2D) and intra- (right Sol-right TA) limb coordination (Figure 2E). The bar graphs presenting the mean polar-plot angles (Figure 2G) and ***r***-values (Figure 2H) for quipazine and 8-OHDPAT show that the mean ***r***-values for intra- and interlimb coordination were significantly higher after 8-OHDPAT than after quipazine applications, suggesting that the 5-HT_7_ receptors participate more significantly in rhythm generation.

**Figure 3:**
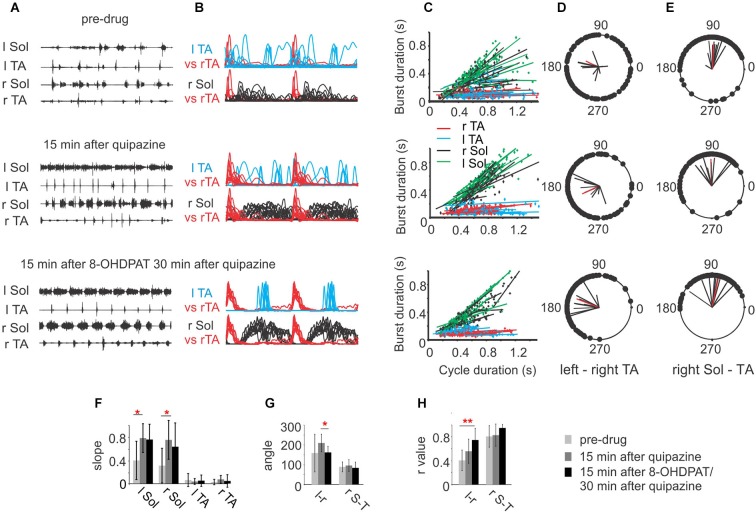
**(A)** EMG activity during hindlimb movements in three sequential experimental conditions: before (upper panel), after a low dose (0.1 mg/kg, i.p.) of quipazine (middle panel) and after a subsequent low dose (0.1 mg/kg, i.p.) of 8-OHDPAT (lower panel). Note the longer but rather irregular burst activity of Sol muscle during locomotor movements after quipazine application and improved regularity after a subsequent dose of 8-OHDPAT. **(B)** Envelopes of EMG burst activity established from raw records rectified, filtered, and normalized to the step cycle (onset of activity in the right TA considered the onset of the cycle); **(C)** regression lines illustrating the relationships between the step cycle durations and burst durations for the left and right TA and Sol muscles for the three conditions; **(D)** and **(E)** polar plots showing the relationships between the onset of r TA activity and either the contralateral TA **(D)** or the ipsilateral r Sol **(E)**. The 0 position on the polar plot corresponds to the onset of activity in the right TA muscle, and the positions of the filled black circles indicate the times of onset of activity in the left TA (interlimb coordination in **D**) or the onset of activity in the right Sol (intralimb coordination in **E**), the black lines each represent results of analysis established for one animal and the red line represents the average results across animals in the analyzed group. **(F)** Bar diagrams showing the mean slopes (±SD) for the regression lines between step cycle durations and burst durations for the left and right TA and Sol muscles in rats in locomotor hindlimb movements in the three experimental conditions. **(G)** Bar diagrams showing the means (±SD) of the angle of the left–right TA (l–r) and of the right Sol–right TA (r S–T) relationships (relative timing of the onsets for the two muscles) represented in each polar plot in the three experimental conditions. **(H)** Bar diagrams showing the means (±SD) of ***r***-value between the times of onset of activity in both left-right and flexor-extensor EMG established for spinal rats (*n* = 5) in the three experimental conditions. Each rat was tested twice with at least a week’s delay between tests. * *P* < 0.05, ** *P* < 0.01, statistical significance established by one-way repeated measures ANOVA followed by Tukey’s *post-hoc* test.

As we demonstrated above, activation of either 5-HT_2_ or 5-HT_7/1A_ receptors alone facilitates hindlimb locomotion in spinal rats, but their effects are significantly different, probably due to activation of different populations of spinal neurons constituting different elements of the spinal cord circuitry that control locomotor movements. Given these differences and our preliminary observation that the combined use of these two drugs results in more robust locomotion without the need for tail stimulation (Sławińska et al., 2012b), we decided to investigate in detail the effects of combined treatment of low doses of 5-HT receptor agonists: quipazine and 8-OHDPAT. As is shown in Figures 3A,B the low dose of quipazine alone significantly prolonged the extensor bursts during the hindlimb movements: however the locomotor pattern remained rather irregular. Addition of 8-OHDPAT improved coordination significantly. An analysis of the relationship between the EMG burst duration and step cycle duration shows significantly increased slopes of regression lines established for the Sol EMG burst activity during a hindlimb locomotor pattern facilitated by quipazine (one-way ANOVA for ***r*** Sol *F*_(2,27)_ = 4.026, *P* = 0.048, for l Sol *F*_(2,27)_ = 5.317, *P* = 0.0157; multiple comparison by Tukey’s *post-hoc* test *P* < 0.05). The slopes were slightly reduced (not significantly) after the subsequent 8-OHDPAT application (Figures 3C,F). Quipazine at a low dose significantly facilitated locomotor hindlimb movements with prolonged extensor activity and plantar stepping; however interlimb coordination (Figures 3D,H) was not significantly improved (before quipazine application ***r*** = 0.401 ± 0.17, while after the drug was applied ***r*** = 0.55 ± 0.20). The difference was not significant (one-way ANOVA *F*_(2,27)_ = 5.931, *P* = 0.019; multiple comparison by Tukey’s *post-hoc* test, *P* > 0.05). Addition of 8-OHDPAT improved interlimb coordination: ***r*** = 0.739 ± 0.19. Although the changes in coordination after addition of 8-OHDPAT were not statistically significant in comparison to the preceding quipazine application alone, when compared with the pre-drug situation interlimb coordination significantly improved (multiple comparison by Tukey’s *post-hoc* test, *P* < 0.01). The need for tail pinching was greatly reduced or eliminated in such cases, consistent with our previous preliminary observation (Sławińska et al., 2012b) after combined drug treatment. Thus, the combined application of both drugs at lower doses produces a markedly improved locomotor outcome. However, the coordination was not improved by the combination of the two drugs in comparison to either drug alone after high dose applications. This result should be taken into account when designing strategies for restoration of locomotion using serotonergic agonists. It should also be noted that 5-HT agonist applications combined with other interventions that promote plantar stepping reveal that there can be an additive effect that degrades locomotion, so the proper balance between drug effect and other effects is necessary (Sławińska et al., 2012b).

**Figure 4:**
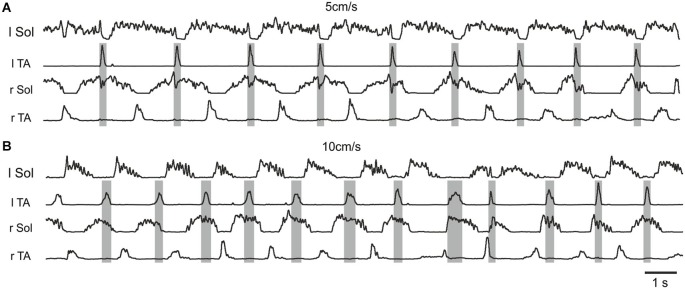
**Examples of rectified and filtered EMG records from left and right Sol and TA muscles during hindlimb locomotor movements after a low dose (0. 1 mg/kg, i.p.) of quipazine and a subsequent low dose (0.1 mg/kg, i.p.) of 8-OHDPAT in a rat suspended over a moving treadmill belt at two speeds: 5 cm/s (A) and 10 cm/s (B)**. Note the presence of a period of inhibition of the r Sol burst that coincided with the presence of the burst of activity in the contralateral flexor EMG (l TA) at the slower speed of locomotion **(A)**, but not during the trial at the higher speed **(B)**.

The presence of a period of inhibition of the soleus burst that coincided with the presence of a burst in the contralateral flexor muscle was consistently observed during the combined drug treatment (Figure 3B third panel, Figure 4). This was confirmed in 5/5 animals. These observations are consistent with excitatory inputs to neurons responsible for production of synchronous movements of the hindlimb, as in gallop, where ipsilateral flexor activity could be associated with inactivation of contralateral extensor activity to some advantage. When the animal is walking on a treadmill with alternating activity in the hindlimbs, this inhibition breaks through and reduces the amplitude of the contralateral extensor burst. We interpret this to be the extensor inhibitory component of a synchrony microcircuit. Examples in Figure 4 taken from one rat demonstrate the presence of a period of inhibition of the r Sol burst that coincided with the presence of the burst of activity in the contralateral flexor EMG (l TA) at the slower speed of locomotion, but not during the trial at the higher speed.

The recent finding that certain commissural neurons involved in the production of hopping gaits in genetically modified mice (Talpalar et al., 2013) are configured to be recruited at different speeds of locomotion prompted us to examine the possibility that such neurons might contribute to the appearance of the synchrony microcircuit resulting from treatment with 5-HT agonists. To this end we examined whether the speed of locomotion could alter the appearance of the synchrony microcircuit. Figure 4 illustrates one example of the results of these experiments, which was a consistent finding in all five cases. Here, the synchrony microcircuit is recruited at the slower speed of locomotion, but not at the higher speed, implicating the V0_V_ excitatory commissural neurons and their target contralateral inhibitory interneurons that terminate on contralateral motoneurons (Menelaou and McLean, 2013; Talpalar et al., 2013) as the basis for this phenomenon.

## Discussion

To summarize the results of the present study, we show here that activation of either 5-HT_2_ or 5-HT_7_/5-HT_1A_ receptors by application of single doses of either quipazine or 8-OHDPAT facilitates activity of the spinal cord network controlling locomotor hindlimb movements in paraplegic rats in the normal horizontal posture. However, markedly different locomotor effects induced by activation of these two receptors suggest that the observed results are related to the activation of different populations of spinal neurons. Activation of 5-HT_2_ receptors led mainly to improvement of extensor muscle activity and less to the improvement of regularity of walking, likely facilitating the output stage of the locomotor system by direct activation of motoneurons. It also may activate the pattern formation interneurons of the locomotor CPG that are the presumed last-order interneurons providing the CPG output to motoneurons. Activation of 5-HT_7_ and 5-HT_1A_ receptors that led to improved left-right alternation in hindlimb walking probably facilitated the activity of the locomotor CPG, without direct actions on the output components of the locomotor system, including motoneurons. These results are consistent with the idea that the spinal cord network responsible for locomotor pattern generation consists of different layers that control motoneuron activity, one the rhythm generator (CPG) and another the pattern formation layer (McCrea and Rybak, 2007, 2008). This hypothesis is supported also by the results of the combined use of the drugs in our investigations, which show that simultaneous activation of different neuronal populations expressing different 5-HT receptors results in production of improved well-coordinated weight supported locomotion. Moreover, the requirement for a smaller dose of both drugs suggests that both neuronal populations play complementary roles in facilitation of locomotor hindlimb movements in spinalized adult rats. In addition, the data reveal the presence of a previously undescribed system of crossed inhibition of ankle extensor muscles that is time-locked to the occurrence of the contralateral flexor burst that is brought into play when neurons that possess receptors activated by quipazine and 8-OHDPAT are recruited. These results are compatible with the activation of neurons that play a role in the production of synchronous activity across the midline, such as occurs in galloping and hopping, and they are strikingly similar to the results obtained in mutant mice showing a role for V0 interneurons commissural microcircuits involved in speed control of hopping (Talpalar et al., 2013).

Chronic treatment with this combination of drugs has been demonstrated to improve locomotion (Antri et al., 2005), and a number of studies have examined the effects of one or the other of these drugs on the restoration of locomotion after spinal cord injury in adult animals (Landry et al., 2006). Combinations of these drugs along with other interventions, such as locomotor training and epidural stimulation, have been reported to improve functional recovery after spinal cord injury (Courtine et al., 2009; Musienko et al., 2011). Many of these studies have used a paradigm in which the rats are placed in an upright posture, a condition that we have shown has its own ability to facilitate recovery of plantar stepping (Sławińska et al., 2012b,c). Here we focus upon the demonstration that different populations of spinal neurons and coordinating microcircuits are recruited by the drugs acting on different 5-HT receptors to account for restoration of plantar stepping without the need for other interventions.

A central issue to the interpretation of our results is which receptors are likely to be involved in the action of these drugs (Hochman et al., 2001). The action of quipazine could be mediated by a few 5-HT receptors for which it has high affinity. These include 5-HT_2A_, 5-HT_2B_ or 5-HT_2C_ receptors. 5-HT_2A_ labeling has been demonstrated to be most intense in motoneurons (Maeshima et al., 1998; Cornea-Hébert et al., 1999; Doly et al., 2004), but it also occurs in other neurons of the ventral horn and in dorsal root ganglion cells. The action of quipazine to facilitate locomotion has been attributed to 5-HT_2A_ receptors (Ung et al., 2008), and 5-HT_2A_ receptors have been implicated in the production of locomotion by stimulation of brainstem serotonergic neurons in neonatal rats (Liu and Jordan, 2005). The affinity of quipazine for 5-HT_2A_ receptors is in the order of four fold higher than for 5-HT_2C_ receptors, and over 50-fold higher than for 5-HT_2B_ receptors.^2^ Another study (Murray et al., 2010) claimed that the appearance of constitutively active 5-HT_2C_ receptors after staggered hemisection accounts for recovery of locomotor function after spinal cord injury. In contrast, after complete transection of the spinal cord (Navarrett et al., 2012) 5-HT_2A_R mRNA expression was upregulated below the site of spinal cord injury, but no changes in 5-HT_2C_R mRNA editing or expression were detected. A similar absence of changes in 5-HT_2C_ receptors below the lesion was reported after contusive injury to the spinal cord (Nakae et al., 2008). It appears possible that the upregulation of constitutively active 5-HT_2C_ receptors as a means of restoring locomotion after spinal cord injury may be limited to the staggered hemisection preparation and/or to the intrathecal application of drugs at the L5-L6 vertebral level (Murray et al., 2010), where locomotor control due to sacrocaudal afferent stimulation (Lev-Tov et al., 2010) might predominate. On the other hand, considerable evidence supports the upregulation of 5-HT_2A_ receptors after complete spinal cord injury, especially in motoneurons (Jordan et al., 2010; Kong et al., 2010, 2011; Navarrett et al., 2012). We have preliminary evidence using intrathecal drug application of antagonists at the lumbar level in intact rats that 5-HT_2A_ but not 5-HT_2C_ receptors are normally involved in the production of locomotion (Sławińska et al., 2012a). Similarly, Fouad et al. (2010) found that blocking constitutive 5-HT_2C_ receptor activity with the potent inverse agonist SB206553 applied intrathecally had no effect on stepping in normal rats.

Motoneuron responses to 5-HT are mediated by a variety of receptors (Schmidt and Jordan, 2000; Heckman et al., 2009; Perrier et al., 2013). The responses are typically an increase in excitability, through several possible mechanisms (*E*_m_ depolarization, reduced AHP, hyperpolarization of voltage threshold, production of persistent inward currents, PICs) when 5-HT_2_ receptors are activated, and depression of motoneuron output when 5-HT_1A_ receptors are activated (reviewed Perrier et al., 2013). It is clear that increased motoneuron excitability is one of the consequences of 5-HT_2_ receptor activation due to quipazine’s actions in our experiments. It seems plausible that the sensory feedback from the improved plantar contact may be at least partially responsible for the increased soleus duty cycle rather than or in addition to direct actions of quipazine on extensor motoneurons. It is known that quipazine can increase the excitability of both flexor and extensor motoneurons (Chopek et al., 2013). Such an increase in flexor motoneuron activity could contribute to the improved placement of the paw and subsequent improved afferent feedback to promote plantar stepping (Sławińska et al., 2012b).

The role of 8-OHDPAT in the production of locomotion appears to be mediated by its action at both 5-HT_7_ and 5-HT_1A_ receptors (Landry et al., 2006), since it could produce locomotion in 5-HT_7_ knockout mice or in the presence of a selective 5-HT_7_ antagonist, and since locomotion could be blocked by antagonists of either 5-HT_7_ or 5-HT_1A_ receptors. The effect of 8-OHDPAT to facilitate locomotion in our experiments appears to be exerted primarily on the rhythm generating and coordinating elements of the CPG. This is indicated by the data showing that the motoneuron excitability is not significantly altered by this drug (see Figure 3; Sławińska et al., 2012b), but its application in low doses together with low doses of quipazine creates a situation where exteroceptive stimulation is no longer necessary to activate the locomotor CPG, and coordination is dramatically improved. This is consistent with the fact that locomotor neurons in the spinal cord possess 5-HT_7_ and 5-HT_1A_ receptors in abundance (Jordan and Schmidt, 2002; Noga et al., 2009). A prominent role for 5-HT_7_ receptors in the control of coordinated muscle activity during locomotion has also been suggested by the investigation of locomotion in 5-HT_7_ receptor knockout mice (Liu et al., 2009) and in experiments using the specific 5-HT_7_ receptor antagonist SB-269970 (Madriaga et al., 2004; Liu and Jordan, 2005; Pearlstein et al., 2005; Liu et al., 2009). Although there is some overlap of 5-HT_2A_ and 5-HT_7_ receptors on locomotor interneurons, there are clearly more 5-HT_7_ positive locomotor neurons than 5-HT_2A_ positive ones (Noga et al., 2009). 5-HT_7_ receptors have not been clearly shown to be associated with motoneurons, due to antibody specificity problems. Almost all locomotor neurons identified by c-fos immunostaining in the above experiments (Jordan and Schmidt, 2002; Noga et al., 2009) were positive for 5-HT_7_ receptors, and a lesser number were positive for 5-HT_2A_ receptors. Faint 5-HT_7_ receptor labeling has been reported in motoneurons (Doly et al., 2005), but we have found the same antibody labels motoneurons in 5-HT_7_ receptor knockout mice.

We conclude from our results and from the information available in the literature about the effects of activation of the various receptors that are the targets of quipazine and 8-OHDPAT that the effects on locomotion that we observe are due largely to actions on 5-HT_2A_ and 5-HT_7_ receptors. We further conclude that the neurons that are activated by 5-HT_2A_ receptors include CPG neurons capable of inducing rhythmic activity, but with incomplete facilitation of coordinating interneurons responsible for inter- and intralimb coordination. Activation of these receptors also results in increased motoneuron excitability and prolonged activity in extensor motoneuron output. The latter effects may be due to the presence of abundant 5-HT_2A_ receptors on motoneurons, especially in order to explain the increased motoneuron excitability. It is more difficult, however, to explain the prolongation of the motoneuron discharge during the stance phase of locomotion on the basis of direct actions of the drug on motoneurons, with the caveat that facilitation of PICs in motoneurons might account for the prolonged activity (Heckman et al., 2009; Perrier et al., 2013). It is also possible that the effects are due in part to the enhancement of activity in CPG neurons of the Pattern Formation layer of the locomotor CPG (McCrea and Rybak, 2007, 2008), which could not only influence the timing of motoneuron activity during the step cycle, but also increase the excitatory drive to motoneurons. If the rhythmic activity produced by quipazine were due to actions directly on motoneurons and on the output stage of the Pattern Formation layer of the CPG, rather than on activation of the Rhythm Generating layer of the CPG, this would explain the incomplete facilitation of intra- and interlimb coordination with this drug. The actions of 5-HT_7_ receptors to trigger rhythmic activity and to activate the neurons responsible for intra- and interlimb coordination, without direct actions on motoneuron excitability, suggests these receptors are located on Rhythm Generation layer neurons as well as on the inhibitory interneurons responsible for coordination (see Figure 2; Jordan and Sławińska, 2011), that are part of the Pattern Formation layer, but without effect on neurons that control the timing of motoneuron activity. This is consistent with the finding that locomotor interneurons may possess either 5-HT_7_ or 5-HT_2A_ receptors, and a smaller proportion possess both (Noga et al., 2009). With this working hypothesis as a basis for further investigation, there are ample opportunities for experiments designed to identify the elements of the CPG responsible for the various functions suggested by our experiments.

Our experiments also provide the first evidence for 5-HT activation of a commissural microcircuit involved in synchronous activation of the hindlimbs (Figures 3, 4). This microcircuit, associated with inhibition of extensor motoneurons at the same time that contralateral flexor motoneurons are active, as well as simultaneous activity in flexor motoneurons bilaterally, may underlie 5-HT modulation of gait. Figures 3, 4 provide the first observation of muscle activity that supports the presence of such a microcircuit, activated by certain 5-HT receptors in adult rat that could contribute to the production of gaits involving synchronous movements of the two hindlimbs, such as hopping and gallop. Only the slightest hints of simultaneous flexor activity were observed in our experiments (Figure 4A), however, suggesting that the neurons excited by the 5-HT agonists are those involved only in the portion of the microcircuit that inhibits contralateral extensors. The findings in Figures 3, 4 suggest that there might be some limitations to the utility of combined treatment with these two agonists for the production of well-coordinated locomotion after spinal cord injury, as we have previously pointed out with respect to the need for a proper balance between drug application and sensory feedback from the sole of the foot (Sławińska et al., 2012b).

The results are consistent with the suggestion that during brainstem evoked locomotion, such as occurs normally, the appropriate coordinating interneurons, including commissural cells, are selected by specific restricted populations of serotonergic neurons to produce the desired gait. In the case of systemic application of serotonergic agonists, this selectivity is lost, so that populations of coordinating commissural cells are activated at inappropriate times.

Serotonergic activation of monosynaptic crossed inhibition might account for our results. Such inhibitory commissural cells terminating on contralateral extensor motoneurons have been demonstrated (Bannatyne et al., 2003). Lamina VIII commissural neurons projecting directly to contralateral ankle extensor motoneurons have been shown to be excited by 5-HT (Hammar et al., 2004). It is clear that many commissural neurons can be excited by 5-HT (Carlin et al., 2006; Zhong et al., 2006a,b; Abbinanti et al., 2012). A disynaptic inhibitory pathway is also a possibility, and some such pathways involve an excitatory commissural cell that activates contralateral Ia inhibitory interneurons (Jankowska et al., 2005).

Another suggestion based on recent work on commissural neuron activity during locomotion involves V0 commissural neurons, which are implicated in the production of a hopping gait. The ipsilateral flexor-related inhibition of contralateral extensor motoneurons could be achieved by selecting for V0_V_ commissural interneurons, which produce contralateral inhibition via a disynaptic pathway at slow speeds of locomotion. When V0_V_ cells predominate, the result is hopping at slow speeds (Menelaou and McLean, 2013; Talpalar et al., 2013). In this scenario the balance between V0_V_ and V0_D_ neurons might be altered under conditions of systemic serotonin agonist application, so that V0_V_ neurons predominate, resulting in the observed inhibition of soleus motoneurons during slow locomotion. This suggests that V0_V_ commissural neurons must receive excitatory input descending serotonergic pathways as well as from excitatory components of the CPG projecting to the contralateral flexors. Our finding that the crossed inhibitory microcircuit activated by 5-HT agonists is apparent during slow but not during faster walking (Figure 4) is consistent with the involvement of V0_V_ neurons in this microcircuit. There is evidence that V0 interneurons possess 5-HT receptors (Dyck et al., 2009; Olsen, 2011), but there have been no direct tests of 5-HT actions on V0 interneurons. Such experiments are warranted by our findings.

The findings described here provide detailed documentation of the differences in EMG activity produced by quipazine and 8-OHDPAT in spinal rats, and make a case for actions of the two drugs on different populations of spinal neurons. An increased understanding of the modes of action of these drugs and the receptors that they activate is of increasing importance because these two drugs have gained prominence in efforts to restore locomotion after spinal cord injury. We also demonstrate that systemic application of these drugs can lead to inappropriate activation of coordinating microcircuits that can interfere with the restoration of the normal locomotor pattern. Further experiments on the involvement of CPG and commissural interneurons in the effects of 5-HT agonists are needed, including recordings from identified interneurons during locomotor activity.

## Author contributions

Urszula Sławińska and Larry M. Jordan conceived, designed research and supervised experiments conducted in Warsaw and in Winnipeg respectively. Urszula Sławińska and Krzysztof Miazga performed research in Warsaw, and Larry M. Jordan and Urszula Sławińska performed research in Winnipeg. Urszula Sławińska, Krzysztof Miazga and Larry M. Jordan analyzed the data and wrote the paper.

## Conflict of interest statement

The authors declare that the research was conducted in the absence of any commercial or financial relationships that could be construed as a potential conflict of interest.

## Footnotes

^1^http://www.scrc.umanitoba.ca/doc/

^2^http://pdsp.med.unc.edu/
